# Association of foetal size and sex with porcine foeto-maternal interface integrin expression

**DOI:** 10.1530/REP-18-0520

**Published:** 2019-01-16

**Authors:** Claire Stenhouse, Charis O Hogg, Cheryl J Ashworth

**Affiliations:** Developmental Biology Division, The Roslin Institute and Royal (Dick) School of Veterinary Studies, University of Edinburgh, Midlothian, UK

## Abstract

Integrins regulate adhesion at the foeto-maternal interface by interacting with secreted phosphoprotein 1 (SPP1) and fibronectin (FN). It is hypothesised that impaired foetal growth of ‘runt’ piglets is linked to altered integrin signalling at the foeto-maternal interface. Placental and endometrial samples associated with the lightest and closest to mean litter weight (CTMLW) (gestational day (GD18, 30, 45, 60 and 90), of both sex (GD30, 45, 60 and 90) (*n* = 5–8 litters/GD), Large White × Landrace conceptuses or foetuses were obtained. The mRNA expression of the integrin subunits (ITG) *ITGA2*, *ITGAV*, *ITGB1*, *ITGB3*, *ITGB5*, *ITGB6*, *ITGB8*, *SPP1* and *FN* was quantified by qPCR. Temporal changes in mRNA expression were observed, with different profiles in the two tissues. Endometrial *ITGB1* (*P* ≤ 0.05, GD45) and *SPP1* (*P* ≤ 0.05, all GD combined and GD60) expression was decreased in samples supplying the lightest compared to the CTMLW foetuses. Placentas supplying female foetuses had decreased expression of *ITGB6* (GD45, *P* ≤ 0.05) and *FN* (GD90, *P* ≤ 0.05) compared to those supplying male foetuses. Endometrial samples supplying females had increased *ITGB3* (*P* ≤ 0.05, GD60) and *FN* (*P* ≤ 0.05, GD30) expression and decreased *SPP1* (*P* ≤ 0.05, GD60) expression compared to male foetuses. Correlations between mean within-gilt mRNA expression and percentage prenatal survival, number of live foetuses or conceptuses and percentage male foetuses were observed. This study has highlighted novel and dynamic associations between foetal size, sex and integrin subunit mRNA expression at the porcine foeto-maternal interface. Further studies should be performed to improve the understanding of the mechanisms behind these novel findings.

## Introduction

Low birthweight infants have an increased risk of mortality and morbidity and can be classified as small for gestational age (SGA) or intra-uterine growth restricted (IUGR). The pig has a high incidence of naturally occurring low birthweight piglets, with many litters having a growth-restricted or ‘runt’ piglet that can weigh less than half of the weight of their largest littermates (Widdowson 1971, Ashworth *et al.* 2001, Wu *et al.* 2010). Considering the high prevalence of low birth weight piglets, it is essential to improve the understanding of the mechanisms governing foetal growth. It is hypothesised that inadequate conceptus attachment to the endometrium contributes to the large variation in piglet weight and high prevalence of low birthweight piglets.

Integrins (ITGs) are glycoprotein transmembrane receptors which exist as heterodimers composed of non-covalently linked alpha (ITGA) and beta (ITGB) subunits (Giancotti & Ruoslahti 1999). Integrins play a central role in cell adhesion and have been shown to be critical in the formation of focal adhesions, cell migration, proliferation and the development of the actin cytoskeleton (Irving & Lala 1995, Schwartz & Assoian 2001, Gallant *et al.* 2005, Delon & Brown 2007). Specific integrin heterodimers can bind to peptides containing an Arg-Gly-Asp (RGD) region, such as secreted phosphoprotein 1 (SPP1, also known as osteopontin (OPN)) and fibronectin (FN). The process of implantation requires significant remodelling of the extracellular matrix at the interface between the conceptus and the mother (Carson *et al.* 2000, White *et al.* 2006), which is believed to be partially regulated by integrin signalling. Both SPP1 (Feinberg *et al.* 1991, Denker 1993, Sutherland *et al.* 1993, Aplin *et al.* 1994, Lessey *et al.* 1994, Ruck *et al.* 1994, Campbell *et al.* 1995, Fassler & Meyer 1995, Schultz & Armant 1995, Stephens *et al.* 1995, Yoshimura *et al.* 1995, Bowen *et al.* 1996, Shiokawa *et al.* 1996, 1999, Fazleabas *et al.* 1997, Schultz *et al.* 1997, Yelich *et al.* 1997, Guillomot 1999, Hodivala-Dilke *et al.* 1999, Johnson *et al.* 1999, 2001, Kimmins & MacLaren 1999, Illera *et al.* 2000, 2003, Bloor *et al.* 2002, Burghardt *et al.* 2002, 2009, Kao *et al.* 2002, Qin *et al.* 2003, Rashev *et al.* 2005, White *et al.* 2005, Zheng *et al.* 2006, Lin *et al.* 2007, Erikson *et al.* 2009*a*, Germeyer *et al.* 2014, Vélez *et al.* 2015, 2017, Frank *et al.* 2017) and FN (Feinberg *et al.* 1991, Schultz & Armant 1995, Bowen *et al.* 1996, Qin *et al.* 2003, Rashev *et al.* 2005, Imakawa *et al.* 2006, Zheng *et al.* 2006, Vélez *et al.* 2015, 2017) have been reported to play a role in early gestation in multiple species by regulating integrin signalling. In the pig, trophoblast and uterine luminal epithelial cells have been shown to express *ITGAV*, *ITGA2*, *ITGA5*, *ITGB1*, *ITGB3* and *ITGB6* (Bowen *et al.* 1996, Erikson *et al.* 2009*a*, Frank *et al.* 2017). It has been suggested that ITGα5β1, ITGαVβ3, ITGαVβ6 and ITGαVβ1 receptors may be able to bind to SPP1, and FN may bind to ITGα5β1, ITGαVβ3 or ITGαIIβ3 (Humphries *et al*. 2006). Further, porcine trophoblast cells have been shown to utilise integrin heterodimers containing ITGAV receptors to bind to SPP1 during implantation (Frank *et al.* 2017).

In addition to a central role in the regulation of implantation and early pregnancy in the pig (Bowen *et al.* 1996, Rashev *et al.* 2005, White *et al.* 2005, Erikson *et al.* 2009*a*, Vélez *et al.* 2015, 2017), integrins and their ligands are expressed at the porcine foeto-maternal interface at multiple gestational days (GD) (Garlow *et al.* 2002, White *et al.* 2005, Hernández *et al.* 2013, Vélez *et al.* 2015, 2017, Frank *et al.* 2017, Steinhauser *et al.* 2017). Considering the non-invasive nature of the porcine placenta (Montiel *et al.* 2013), and the substantial remodelling that must occur to ensure adequate nutrient transfer to the foetus (Vallet & Freking 2007), it is unsurprising that adhesive processes must be present throughout gestation to maintain a successful pregnancy.

It is hypothesised that the mRNA expression of integrin subunits, *FN* and *SPP1*, will be decreased in placental and endometrial samples associated with the lightest conceptuses or foetuses compared to their normal-sized littermates throughout gestation. To test this hypothesis, samples were compared within litter at five GDs of interest.

It has previously been demonstrated that significant variation in foetal size can be observed at GD30 (Pettigrew *et al.* 1986, Wise *et al.* 1997, Finch *et al.* 2002, Foxcroft & Town 2004, Foxcroft *et al.* 2006), which is thought to be reflective of the postnatal within-litter variation in the piglet size observed. Therefore, placental and endometrial samples from early gestation were utilised as part of this study. During pregnancy, temporal changes in the rate of foetal and placental growth can be observed (Marrable 1971). After the initially fast period of placental growth, the growth rate of the porcine placenta plateaus in mid-gestation, whereby instead of continuing to increase in weight, the structure of the placenta undergoes extensive remodelling to increase the surface area available for nutrient exchange with the developing foetus (Vallet & Freking 2007). Whilst the placental growth rate is decreased, the foetus is undergoing rapid growth, placing large demands upon the placenta. This study has utilised placental and endometrial samples from GD18, 30, 45, 60 and 90, which were selected to allow investigation of these tissues throughout gestation due to the dynamic relationship between foetal and placental growth at the foeto-maternal interface.

Recent literature has suggested that sexual dimorphism in placental development exists in humans (Rosenfeld 2015, Kalisch-Smith *et al.* 2017), which may translate to the observed sexual dimorphism in phenotype in response to adverse conditions during pregnancy. In the pig, it has been suggested that males are at a disadvantage compared to their female littermates post-natally (Baxter *et al.* 2012). Further, recent investigations in our laboratory have reported sexual dimorphism in both placental and endometrial vascularity in the pig (Stenhouse 2018, Stenhouse *et al.* 2018*a*). Considering this, it is hypothesised that decreased expression of the integrin receptors and their ligands will be observed in placental and endometrial samples supplying male foetuses compared to their female littermates.

The number of live born piglets per year is an economically important factor for the pig industry (Koketsu *et al.* 2017). Further, the sex ratio of the litter is known to influence reproductive success (Lamberson *et al.* 1988, Drickamer *et al.* 1997, 1999, Huhn *et al.* 2002, Górecki 2003). Considering this, and the proposed role of integrin signalling at the foeto-maternal interface, the relationship between integrin mRNA expression and percentage prenatal survival, number of live foetuses and the percentage of male foetuses in the litter was investigated.

## Materials and methods

All procedures were performed with approval from The Roslin Institute (University of Edinburgh) Animal Welfare and Ethical Review Board and in accordance with the U.K. Animals (Scientific Procedures) Act, 1986.

### Experimental animals and sample collection

Large White × Landrace gilts (age 11–14 months; *n* = 31) were observed daily for signs of oestrus and were housed in groups of 6–8 animals per pen. Oestrous cyclicity and ovarian function were controlled in accordance with routine normal practice at The Roslin Institute Large Animal Unit. In a subset of gilts (distribution between the GD investigated indicated in Supplementary Table 1, see section on supplementary data given at the end of this article) oestrus was synchronised by daily feeding of 20 mg Altrenogest (Regumate, Hoechst Roussel Vet Ltd.) for 18 days followed by injection of pregnant mare serum gonadotrophin (PMSG; Intervet UK Ltd) and human chorionic gonadotrophin (hCG; Intervet UK Ltd) (Stenhouse *et al.* 2018*a*). All gilts were inseminated twice daily for the duration of oestrus with semen from one of four Large White boars. The boars used were equally distributed throughout the GD to minimise any effect of sire. The first day of insemination was assigned as GD0 and samples were obtained at GD18, 30, 45, 60 and 90. Gilts were killed at the GD of interest with sodium pentobarbitone 20% w/v (Henry Schein Animal Health) at a dose of 0.4 mL/kg by intravenous injection via a cannula inserted in the ear vein. Following confirmation of death, mid-ventral incision revealed the reproductive tract. The tract was lifted from the body cavity and placed in a dissecting tray. Both uterine horns were dissected, from the ovary towards the cervix. The uterine lumen was occluded between each foeto-placental unit by tying with string to ensure that the tissue associated with particular conceptuses or foetuses could be identified later.

At GD18, the uterine tract was rinsed with saline and a string was used to tie the end of the right and left uterine horns at the bifurcation. The uterine horns were cut between the two pieces of string and each uterine horn was placed in a floatation device. The device contained a solution to preserve the integrity of the RNA (700 g ammonium sulphate (SLS) was dissolved in 935 mL of RNase-free water with heat and stirring). Once dissolved, 25 mL of 1 M sodium citrate (Fisher Scientific) and 40 mL of 0.5 M EDTA were added. The solution was adjusted to pH 5.2 using concentrated sulphuric acid and stored at room temperature until required. Using dissection scissors, the uterine horn was opened along the mesometrial side, and the conceptuses floated in the solution. Individual conceptuses were removed from the floatation device with forceps and weighed in a cryovial (Starlab). The uterine lumen was occluded between each conceptus to ensure that endometrial samples associated with particular conceptuses could be identified. The lightest and CTMLW conceptus was identified based on weight and snap-frozen in liquid nitrogen and stored at −80 °C for RNA extraction.

On the remaining GD investigated, foetuses were identified as ‘live’ or ‘dead’ based on their morphology at the time of dissection and were weighed. At GD45, 60 and 90, foetal sex was determined morphologically. DNA was isolated from the GD30 foetuses using the DNeasy Blood and Tissue DNA extraction kit (Qiagen), and PCR was performed for the sex-determining region Y (Sry) – a region of the Y chromosome – as previously described (Stenhouse *et al.* 2018*b*) to determine the sex of the foetuses. The lightest and CTMLW foetuses (GD30), of both sex (GD45, 60 and 90), were identified based on foetal weight. From the anti-mesometrial side, placental and endometrial samples were taken from each foeto-placental unit of interest, snap-frozen in liquid nitrogen and stored at −80 °C for RNA extraction.

### Analysis of mRNA expression by qPCR

#### Total RNA extraction and cDNA synthesis

RNA was extracted from 20 to 50 µg of snap-frozen placental and endometrial samples as previously described (Stenhouse *et al.* 2018*a*). The RNA was quantified, and the quality was assessed spectrophotometrically using a Nanodrop ND-1000 (Labtech International Ltd.) and electrophoretically using a Tapestation 2200 (Agilent Technologies). The mean A260/A280 and RNA Integrity Number Equivalent (RINe) for samples within each GD are detailed in Supplementary Table 2. Extracted RNA was stored at −80 °C until required. If the RINe value obtained remained lower than the ranges detailed in Supplementary Table 2, the sample was excluded from the analyses.

cDNA was prepared from 0.3 µg of RNA with SuperScript III reverse transcriptase (Life Technologies) following the manufacturer’s instructions. Each reaction contained 250 ng random primers (Promega) and 40 units RNaseIn (Promega). Negative controls without reverse transcriptase were included to check for genomic contamination. Reverse transcription was performed in duplicate for each sample and pooled, and the cDNA was stored at −20 °C until required.

#### Relative expression of candidate genes

Quantitative PCR was performed on a Stratagene MX3000 instrument using Platinum SYBR Green SuperMixUTG (Life Technologies) using cDNA from placental (GD30, 45, 60 and 90; *n* = 6, 6, 6 and 8 litters respectively) and endometrial (GD18, 30, 45, 60 and 90; *n* = 5, 5, 6, 6 and 6 litters respectively) samples. The samples were associated with the lightest and CTMLW conceptuses or foetuses at GD18 and 30, and the lightest and CTMLW foetuses of both sex at GD45, 60 and 90. The final concentrations of magnesium, ROX reference dye and each primer were 3 mM, 50 nM and 400 nM respectively in a 25 µL reaction volume. All qPCRs were carried out at an annealing temperature of 60 °C and dissociation curves consisting of single peaks were generated. The mRNA expression of *ITGA2*, *ITGAV*, *ITGB1*, *ITGB3*, *ITGB5*, *ITGB6*, *ITGB8*, *SPP1* and *FN* was quantified in both tissues (King *et al.* 2011, Hernández *et al.* 2013, Frank *et al.* 2017). Appropriate reference genes were identified by analysis of 11 candidate reference genes (Erkens *et al.* 2006, Nygard *et al.* 2007) using geNORM V3.5 (Ghent University Hospital, Centre for Medical Genetics). The reference genes *TBP1* (TATA box-binding protein) and *HPRT1* (hypoxanthine phosphoribosyltransferase 1) were utilised to normalise placental mRNA expression, and endometrial mRNA expression was normalised using the reference genes *TBP1*, *YWHAZ* (Tyrosine 3-monooxygenase/tryptophan 5-monooxygenase activation protein and zeta polypeptide) and *TOP2B* (Topoisomerase II beta). The primer sequences for all genes are detailed in Supplementary Table 3.

Serial dilutions of pooled cDNA ranging from 1:5 to 1:640 in nuclease-free water were used as standards. Sample cDNA was diluted 1:25 and 5 µL of diluted sample, standard or control were added per well. Each plate contained duplicate wells of a no template control, standards, sample cDNA and reverse transcriptase blanks. Data were analysed using qbase+ software V3.0 (Biogazelle). A target- and run-specific strategy was employed and the results, normalised to the two reference genes, were scaled to the minimum sample. The mean slope, intercept, PCR efficiency and *R* ^2^ values are detailed in Supplementary Table 4.

### Statistical analysis

All statistical analyses were performed using Minitab 17 or GenStat 13.1 (VSN International Ltd.). The normalised mean value for each placental and endometrial sample was taken and the normality of the distribution of the data was assessed by an Anderson-Darling test. If a *P* value of ≤0.05 was obtained, then the data were not considered to have a normal distribution. Outliers were tested for using a Grubbs outlier test and were excluded systematically, with normality within each group being reassessed following each exclusion. Log_10_ transformations were carried out where appropriate to improve the normality of the distribution of the data. The effect of foetal size was assessed by comparing values for the true lightest and true CTMLW at GD18, 30, 45, 60 and 90. Effects of foetal sex were assessed by comparing values from placental and endometrial samples supplying foetuses of both sex at GD30, 45, 60 and 90. Where data had a normal distribution, ANOVA for GD, foetal size or sex was performed, with a block for gilt to account for the common maternal environment. A *post hoc* Tukey test was performed where appropriate. Where data did not have a normal distribution, Kruskal–Wallis and Mann Whitney tests were performed. Within gilt, the mean expression for each gene of interest was calculated within tissue. Pearson’s correlations were performed within GD to determine the association between the mean gene expression within gilt and the percentage of males in the litter (sex ratio), percentage prenatal survival (calculated by dividing the number of live foetuses by the number of *corpora lutea*, multiplied by 100) and the number of live foetuses. In all experiments, results were considered significant when *P* ≤ 0.05, tending towards significant when *P* was >0.05 and <0.1 and not significant when *P* ≥ 0.1.

## Results

### Temporal changes in integrin mRNA expression

**Figure 1:**
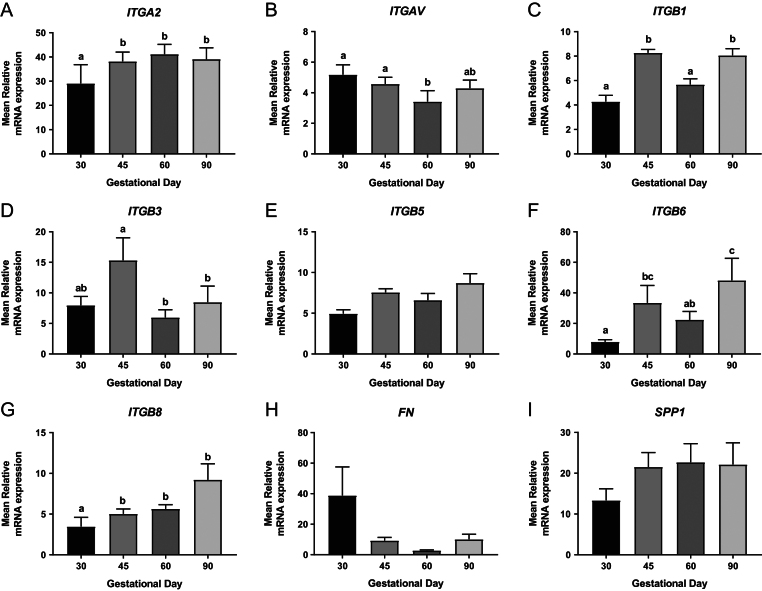
Integrin mRNA expression in placental tissues on days 30, 45, 60 and 90 of pregnancy. mRNA expression of *ITGA2* (A), *ITGAV* (B), *ITGB1* (C), *ITGB3* (D), *ITGB5* (E), *ITGB6* (F), *ITGB8* (G), *FN* (H) and *SPP1* (I) in placental samples at GD30, 45, 60 and 90. Error bars represent s.e.m. *n* = 9–23 foetuses/GD.

No significant day effect was observed in the placental expression of *ITGB5* or *SPP1* (Fig. 1E and I). An overall day effect was observed in the placental expression of *ITGA2* (*P* ≤ 0.05; Fig. 1A), with GD30 placentas having decreased expression compared to the other GD investigated. Placental *ITGAV* expression was influenced by GD (*P* ≤ 0.05; Fig. 1B), with decreased expression observed at GD60 compared to GD30 and GD45. An overall GD effect was observed in the placental expression of *ITGB1* (*P* ≤ 0.001; Fig. 1C), with increased expression observed at GD45 and GD90 compared to GD30 and GD60. Placental *ITGB3* expression was decreased at GD60 and GD90 compared to GD45 (*P* ≤ 0.05; Fig. 1D). Temporal changes in the placental expression of *ITGB6* were observed (*P* ≤ 0.01; Fig. 1F), with low placental expression at GD30, which significantly increased at GD45. Placental *ITGB6* expression then significantly increased to its peak expression level at GD90. Placental *ITGB8* expression increased with advancing gestation (*P* ≤ 0.01; Fig. 1G), with a significant increase observed between GD30 and GD45. Placental *FN* expression varied throughout gestation (*P* ≤ 0.01; Fig. 1H).

**Figure 2:**
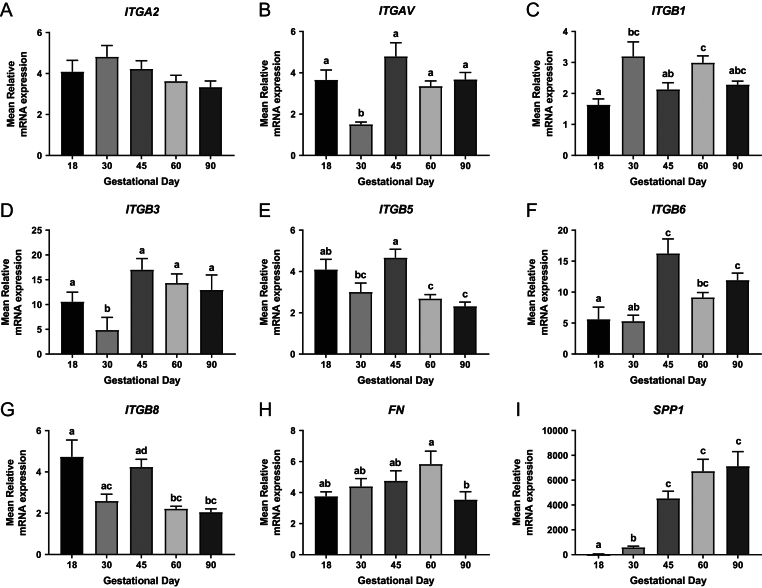
Integrin mRNA expression in endometrial tissues on days 18, 30, 45, 60 and 90 of pregnancy. mRNA expression of *ITGA2* (A), *ITGAV* (B), *ITGB1* (C), *ITGB3* (D), *ITGB5* (E), *ITGB6* (F), *ITGB8* (G), *FN* (H) and *SPP1* (I) in endometrial samples at GD18, 30, 45, 60 and 90. Error bars represent s.e.m. *n* = 9–30 foetuses/GD.

An overall GD effect was observed in *ITGA2* endometrial expression (*P* ≤ 0.05; Fig. 2A). The endometrial expression of *ITGAV* (*P* ≤ 0.001; Fig. 2B) and *ITGB3* (*P* ≤ 0.01; Fig. 2D) were decreased at GD30 compared to the other GD investigated. During gestation, *ITGB1* (*P* ≤ 0.01; Fig. 2C) and *ITGB8* (*P* ≤ 0.001; Fig. 2G) endometrial expression fluctuated. Endometrial *ITGB5* expression also fluctuated throughout gestation (*P* ≤ 0.001; Fig. 2E), with decreased expression observed at GD30, GD60 and GD90 compared to GD18 and GD45. *ITGB6* endometrial expression was increased in mid and late gestation compared to early gestation, with a notable increase in expression observed between GD30 and GD45 (*P* ≤ 0.001; Fig. 2F). Endometrial *SPP1* expression increased with advancing gestation (*P* ≤ 0.001; Fig. 2I).

### Associations between foetal size and integrin mRNA expression

**Figure 3:**
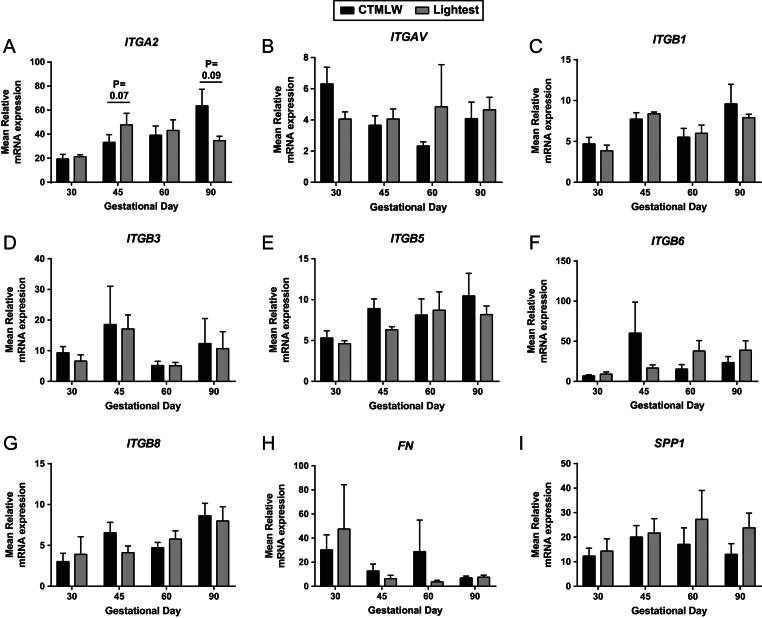
Integrin mRNA expression in placental tissues associated with the lightest and CTMLW conceptuses and foetuses on days 30, 45, 60 and 90 of pregnancy. mRNA expression of *ITGA2* (A), *ITGAV* (B), *ITGB1* (C), *ITGB3* (D), *ITGB5* (E), *ITGB6* (F), *ITGB8* (G), *FN* (H) and *SPP1* (I) in placental samples associated with the lightest and CTMLW foetuses at GD30, 45, 60 and 90. Error bars represent s.e.m. *n* = 4–7 foetuses/GD.

No statistically significant associations between foetal size and placental expression of *ITGAV* (Fig. 3B), *ITGB1* (Fig. 3C), *ITGB3* (Fig. 3D), *ITGB5* (Fig. 3E), *ITGB6* (Fig. 3F), *ITGB8* (Fig. 3G), *FN* (Fig. 3H) or *SPP1* (Fig. 3I) were observed. At GD45 (*P* = 0.07; Fig. 3A), placental samples supplying the lightest foetuses tended to have increased *ITGA2* expression compared to those supplying the CTMLW foetuses. The direction of this difference switched at GD90 (*P* = 0.09; Fig. 3A), with placental samples supplying the lightest foetuses having decreased *ITGA2* expression compared to those supplying the CTMLW foetuses.

**Figure 4:**
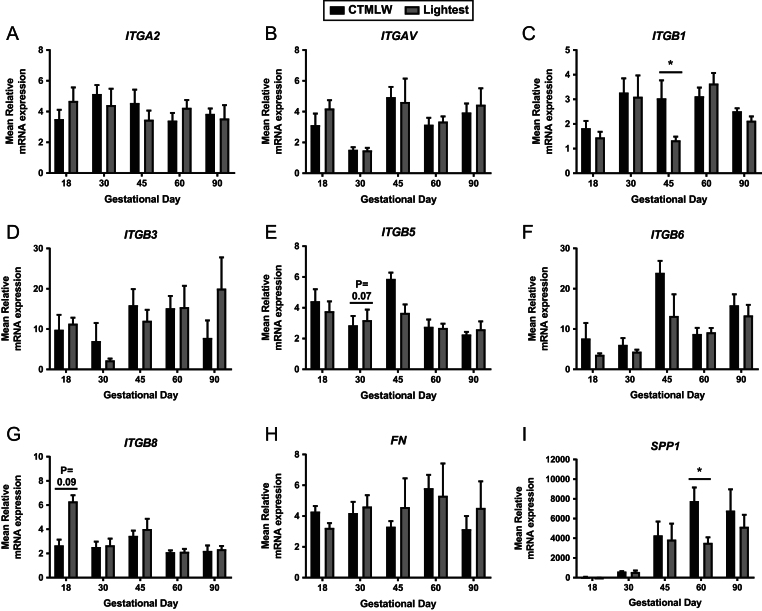
Integrin mRNA expression in endometrial tissues associated with the lightest and CTMLW conceptuses and foetuses on days 18, 30, 45, 60 and 90 of pregnancy. mRNA expression of *ITGA2* (A), *ITGAV* (B), *ITGB1* (C), *ITGB3* (D), *ITGB5* (E), *ITGB6* (F), *ITGB8* (G), *FN* (H) and *SPP1* (I) in endometrial samples associated with the lightest and CTMLW conceptuses and foetuses at GD18, 30, 45, 60 and 90. Error bars represent s.e.m. *P ≤ 0.05. *n* = 3–8 foetuses/GD.

No statistically significant associations between conceptus or foetal size and endometrial expression of *ITGA2* (Fig. 4A), *ITGAV* (Fig. 4B), *ITGB3* (Fig. 4D), *ITGB5* (Fig. 4E), *ITGB6* (Fig. 4F) or *FN* (Fig. 4H) were observed. Endometrial *ITGB1* expression was decreased in samples supplying the lightest foetuses compared to those supplying the CTMLW foetuses at GD45 (*P* ≤ 0.05; Fig. 4C). At GD30, a trend towards endometrial samples supplying the lightest foetuses having increased *ITGB5* expression compared to samples supplying the CTMLW foetuses was observed (*P* = 0.07; Fig. 4E). Further, endometrial samples associated with the lightest conceptuses tended (*P* = 0.09; Fig. 4G) to have increased *ITGB8* expression compared to the CTMLW conceptuses at GD18. An overall size effect in *SPP1* expression was observed (*P* ≤ 0.05; Fig. 4I), with a statistically significant decrease in expression observed in endometrial samples supplying the lightest foetuses compared to the CTMLW foetuses at GD60 (*P* ≤ 0.05).

### Associations between foetal sex and integrin mRNA expression

**Figure 5:**
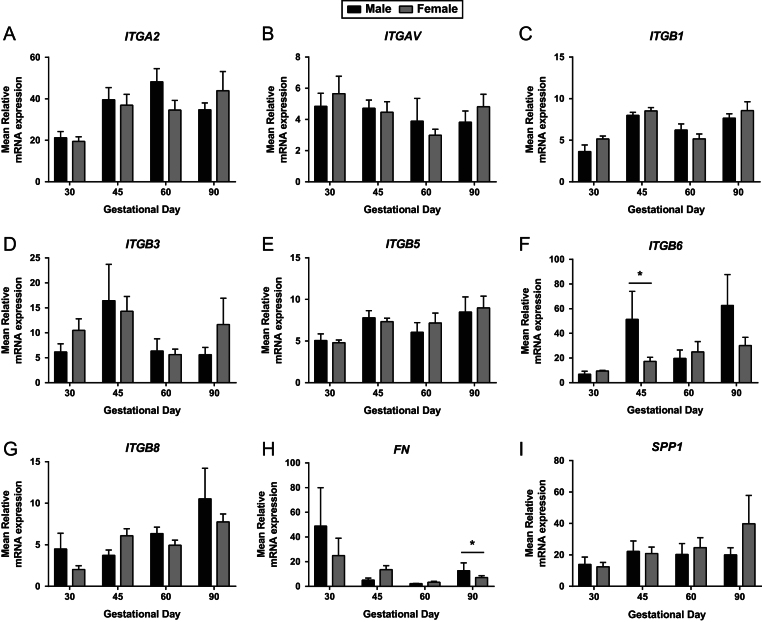
Integrin mRNA expression in placental tissues associated with male and female foetuses on days 30, 45, 60 and 90 of pregnancy. mRNA expression of *ITGA2* (A), *ITGAV* (B), *ITGB1* (C), *ITGB3* (D), *ITGB5* (E), *ITGB6* (F), *ITGB8* (G), *FN* (H) and *SPP1* (I) in placental samples associated with male and female foetuses at GD30, 45, 60 and 90. Error bars represent s.e.m. *P ≤ 0.05. *n* = 4–12 foetuses/GD.

No statistically significant associations between foetal sex and placental expression of *ITGA2* (Fig. 5A), *ITGAV* (Fig. 5B), *ITGB1* (Fig. 5C), *ITGB3* (Fig. 5D), *ITGB5* (Fig. 5E), *ITGB8* (Fig. 5G) or *SPP1* (Fig. 5I) were observed. Significant associations between foetal sex and *ITGB6* (GD45 *P* ≤ 0.05; Fig. 5F) and *FN* (GD90 *P* ≤ 0.05; Fig. 5H) expression were observed, with placentas associated with female foetuses having decreased expression compared to those associated with male foetuses.

**Figure 6:**
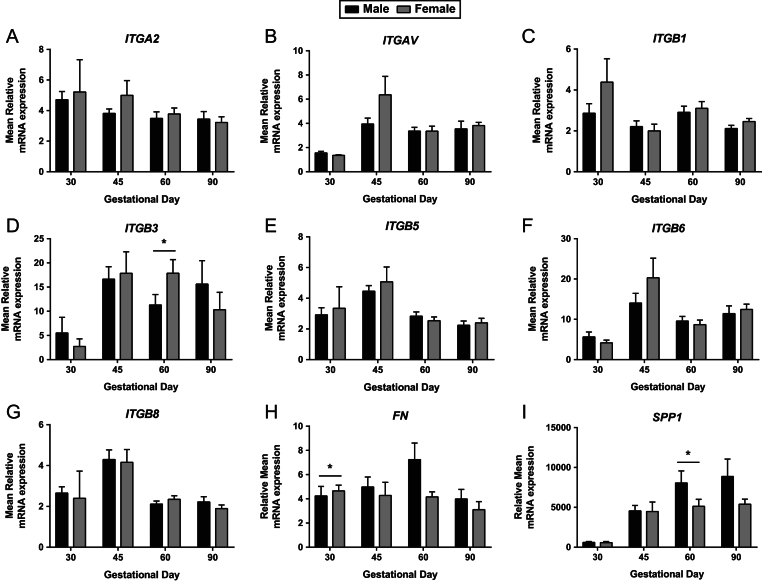
Integrin mRNA expression in endometrial tissues associated with male and female foetuses on days 30, 45, 60 and 90 of pregnancy. mRNA expression of *ITGA2* (A), *ITGAV* (B), *ITGB1* (C), *ITGB3* (D), *ITGB5* (E), *ITGB6* (F), *ITGB8* (G), *FN* (H) and *SPP1* (I) in endometrial samples associated with male and female foetuses at GD30, 45, 60 and 90. Error bars represent s.e.m. *P ≤ 0.05. *n* = 3–16 foetuses/GD.

No statistically significant associations between foetal sex and endometrial expression of *ITGA2* (Fig. 6A), *ITGAV* (Fig. 6B), *ITGB1* (Fig. 6C), *ITGB5* (Fig. 6E), *ITGB6* (Fig. 6F) or *ITGB8* (Fig. 6G) were observed. Endometrial samples supplying females at GD60 had increased *ITGB3* expression compared to those supplying male foetuses (*P* ≤ 0.05; Fig. 6D). At GD30, *FN* expression was increased in endometrial samples supplying females compared to those supplying male foetuses (*P* ≤ 0.05; Fig. 6H). *SPP1* endometrial expression was decreased in samples supplying female foetuses compared to their male littermates at GD60 (*P* ≤ 0.05; Fig. 6I).

### Associations between mean gilt placental and endometrial integrin expression and litter characteristics

At GD30, an inverse correlation between mean placental *ITGB1* expression and percentage of males in the litter was observed (*P* ≤ 0.01; Table 1). Placental *ITGAV* (GD60 and GD90 *P* ≤ 0.05), *ITGB5* (GD90 *P* ≤ 0.001), *ITGB6* (GD90 *P* ≤ 0.01), *ITGB8* (GD90 *P* ≤ 0.01) and *FN* (GD90 *P* ≤ 0.001) expression were positively correlated with the number of live foetuses (Table 1).

**Table 1:** Significant correlations between integrin mRNA expression and litter characteristics.

Gestational day	Tissue	Gene	Variable	RSq (%)	*P* value	Number XY pairs	Positive/Negative
30	Placenta	*ITGB1*	Percentage male foetuses	85.9	≤0.01	6	Negative
60	Placenta	*ITGAV*	Number of live foetuses	64.6	≤0.05	6	Positive
90	Placenta	*ITGAV*	Number of live foetuses	70.3	≤0.05	7	Positive
90	Placenta	*ITGB5*	Number of live foetuses	90.0	≤0.001	7	Positive
90	Placenta	*ITGB6*	Number of live foetuses	74.0	≤0.01	7	Positive
90	Placenta	*ITGB8*	Number of live foetuses	76.2	≤0.01	7	Positive
90	Placenta	*FN*	Number of live foetuses	89.3	≤0.001	7	Positive
18	Endometrium	*SPP1*	Percentage prenatal survival	79.7	≤0.05	5	Positive
18	Endometrium	*ITGB5*	Number of live conceptuses	94.7	≤0.01	5	Negative
30	Endometrium	*ITGB8*	Number of live foetuses	91.1	≤0.05	4	Negative
45	Endometrium	*ITGB6*	Percentage male foetuses	77.9	≤0.05	6	Positive
45	Endometrium	*ITGAV*	Percentage prenatal survival	84.3	≤0.01	6	Positive
90	Endometrium	*FN*	Percentage male foetuses	73.1	≤0.05	6	Positive

A positive correlation between both mean endometrial *ITGB6* (GD45 *P* ≤ 0.05) and* FN* (GD90 *P* ≤ 0.05) expression and the percentage of males was observed (Table 1). Both endometrial *SPP1* (GD18 *P* ≤ 0.05) and *ITGAV* (GD45 *P* ≤ 0.01) expression were positively correlated with percentage prenatal survival (Table 1). Endometrial *ITGB5* (GD18 *P* ≤ 0.01) and *ITGB8* (GD30 *P* ≤ 0.05) were inversely correlated with the number of live conceptuses or foetuses (Table 1).

## Discussion

Integrin signalling has been heavily implicated in the establishment and maintenance of pregnancy by regulating adhesion at the foeto-maternal interface. This study demonstrated an association between mRNA expression of integrins and their ligands, and foetal size at the porcine foeto-maternal interface. There were also novel relationships between foetal sex and both endometrial and placental mRNA expression. Intriguingly, differences in gene expression observed in placental and endometrial tissue appear to occur independently of one another.

This study identified novel associations between foetal size and the endometrial expression of *ITGB1* and *SPP1*. In humans, it has been shown that extravillous trophoblast cells in term placentas which supplied IUGR infants had decreased expression of ITGα2β1, ITGα3β1 and ITGα5β1 integrin receptors compared to those which supplied normally grown infants (Zygmunt *et al.* 1997). In the current study, endometrial samples supplying the lightest foetuses had decreased *ITGB1* expression at GD45 compared to those supplying their normal-sized littermates, reinforcing the suggestion ITGB1 and its ligands may have a role in the regulation of foetal growth in the pig. Further, the ITGα5β1 receptor has been suggested to bind both FN and SPP1 at the foeto-maternal interface in the pig (Frank *et al.* 2017). Whilst no decrease in the expression of *SPP1* or *FN* were observed in the endometrial samples supplying the lightest foetuses compared to their normal-sized littermates at GD45, decreased endometrial *SPP1* expression was observed at GD60.

Emerging evidence in humans suggests that sexual dimorphism in placental development is responsible for sexual differences to disease susceptibility post-natally (Rosenfeld 2015, Kalisch-Smith *et al.* 2017). It has been proposed that male new-born piglets have a survival disadvantage compared to their female littermates (Baxter *et al.* 2012). However, whether this difference arises prenatally due to sexual dimorphism in placental development has not been determined in the pig. Currently, the suggestion of sexual dimorphism in placental or endometrial integrin expression in any species has not been explored. However, in a recent RNA sequencing experiment using human placentas from 10.5 to 13.5 weeks, it was identified that placentas supplying female foetuses had increased *ITGB8* expression compared to those supplying males (Gonzalez *et al.* 2018).

Endometrial *FN* and *ITGB3* expression were increased in samples associated with female foetuses compared with their male littermates at GD30 and 60 respectively. The differential expression of *FN* in the endometrium but not the placenta at this early stage of gestation may indicate the presence of differential signalling between the conceptus and the endometrium in early gestation, although the mechanisms behind this require further investigation.

An inverse relationship between the percentage of males in the litter and placental *ITGB1* (GD30) expression and positive correlations between both endometrial *ITGB6* (GD45) and *FN* (GD90) expression and the percentage of males in the litter were observed. Intriguingly, whilst these results do not mirror the sex differences observed within tissue within GD, placental* ITGB6* and* FN* were demonstrated to be associated with foetal sex at GD45 and GD90 respectively. Interestingly, endometrial *ITGB5* and *ITGB8* expression were inversely correlated with the number of live conceptuses or foetuses in early gestation, whereas in late gestation placental *ITGAV*, *ITGB5*, *ITGB6*, *ITGB8* and *FN* expression were positively correlated with the number of live foetuses. The width of the folded bilayer at the foeto-maternal interface is known to increase significantly from GD65 to GD105 to increase the available surface area for exchange, allowing adequate nutrient transfer to meet the demands of the exponentially growing foetus (Vallet & Freking 2007). Further, there is some evidence to suggest that as litter size increases, uterine capacity becomes a limiting factor (Ford *et al.* 2002, Vallet *et al.* 2014), resulting in smaller placentas. It is proposed that increased remodelling of the interface must occur to attempt to improve placental efficiency and maintain adequate nutrient transfer to the developing foetus. The observed increase in gene expression in late gestation would suggest increased cell adhesion during this period of remodelling. These novel correlations between integrin mRNA expression and number of live foetuses, percentage prenatal survival and percentage of male foetuses in the litter which, considering the importance of these characteristics post-natally, may be of interest to investigate further.

Importantly, this study has demonstrated that endometrial* SPP1* expression was associated with both foetal size and sex, suggesting the actions of SPP1 at the foeto-maternal interface are associated with foetal development. Binding of SPP1 to the ITGAVB3 receptor has been demonstrated to activate the Akt (Protein Kinase B)/eNOS (endothelial nitric oxide synthase) signalling pathway, leading to increased proliferation, migration and tube formation of endothelial cells *in vitro* (Dai *et al.* 2009, Wang *et al.* 2011). Dunlap *et al.* (2008) demonstrated in the sheep a relationship between uterine von Willebrand Factor (vWF) staining and SPP1, and that uterine arterial endothelial cells produce SPP1 during angiogenesis *in vitro*. Analysis of GD9, 12 and 15 porcine endometrial samples identified the presence of a 32kDA fragment of SPP1, which is known to bind to the cell surface of endothelial cells (Erikson *et al.* 2009*b*, Bayless *et al.* 2010). In these studies, *in vitro* experiments using blood samples from new-born piglets demonstrated that SPP1 has a positive effect on the migration and adhesion of endothelial cells, suggesting a potential role in regulating placental and endometrial angiogenesis. At the pig foeto-maternal interface, there are two keys stages of gestation when angiogenesis occurs. The first is at ~GD13-18 (Keys *et al.* 1986), which corresponds to the period of conceptus attachment, and the second wave of angiogenesis occurs at approximately GD50 (Vonnahme *et al.* 2001), which corresponds to the period when placental growth is beginning to plateau and the foetus is about to undergo exponential growth. An additional study utilising the same samples used in the present study revealed striking associations between foetal size and sex and angiogenesis at the foeto-maternal interface, which were dependent on the GD investigated (Stenhouse 2018a, b). Therefore, considering the role of SPP1 and ITGAVB3 during implantation (Erikson *et al.* 2009*a*, Frank *et al.* 2017) and the results of the current study, it could be hypothesised that SPP1 has an additional role in inducing altered angiogenesis in foetuses of different size and sex which should be investigated further.

## Conclusion

A comprehensive temporal analysis of the mRNA expression of key integrin subunits, *FN* and* SPP1* throughout gestation in both placental and endometrial samples was performed, identifying previously undescribed temporal changes in integrin expression. Endometrial expression of *ITGB1* and *SPP1* was decreased in samples associated with the lightest foetuses compared to samples supplying their normally grown littermates at GD45 and 60 respectively. Overall, this demonstrated that foetal size is related to integrin signalling at the foeto-maternal interface but surprisingly this appears to occur in a temporal manner. Novel associations between foetal sex and the expression of *ITGB3*, *ITGB6*,* SPP1* and *FN* at the foeto-maternal interface were also observed. The results presented in this study are intriguing and the mechanisms behind these differences warrant further investigation, especially during preimplantation development and early pregnancy.

## Supplementary Material

Supplementary Table 1

Supplementary Table 2

Supplementary Table 3

Supplementary Table 4

## Declaration of interest

The authors declare that there is no conflict of interest that could be perceived as prejudicing the impartiality of the research reported.

## Funding

The Roslin Institute receives Institute Strategic Grant funding from the BBSRC (BB/J004316/1). C S was in receipt of a studentship from the University of Edinburgh.
