# Supplementary material for: Association of foetal size and sex with porcine foeto-maternal interface integrin expression
Source: Reproduction. 2019 Jan 16;157(4):317–28. doi: 10.1530/REP-18-0520 (PMC6391912; doi:10.1530/REP-18-0520)
Supplement: Supplementary Table 1 [file supplementary_table_1.pdf]

Supplementary Table 1: Summary of Litter Characteristics

Parameter	GD18 (n=5) (PMSG=1/5)		GD30 (n=6) (PMSG=3/6)		GD45 (n=6) (PMSG=1/6)		GD60 (n=6) (PMSG=3/6)		GD90 (n=8) (PMSG=3/8)	
	Mean ± S.E.M.	Range	Mean ± S.E.M.	Range	Mean ± S.E.M.	Range	Mean ± S.E.M.	Range	Mean ± S.E.M.	Range
Gestational Day (GD)	18.4 ± 0.245	18-19	30	n/a	45 ± 0.516	43 - 46	60.5 ± 0.563	58 - 62	90.375 ± 0.324	89 - 92
Ovulation Rate	31.6 ± 9.678	19-70	18.833 ± 1.701	13 - 25	21.5 ± 2.814	14 - 33	22.667± 5.395	14 - 49	22.375 ± 2.345	15 - 37
Litter Size	26 ± 5.367	11-44	11.5 ± 2.513	6-15	16.5 ± 1.455	12 - 20	12.667 ± 1.430	9 - 19	14.375 ± 0.844	13 - 20
Prenatal Survival (%)	94.559 ± 19.436	42.308-152.632	60.315 ± 10.837	35.294 - 100	80.382 ± 7.599	57.143 - 93.333	62.412± 7.451	36.735 - 86.666	67.044 ± 5.215	54.054 - 93.333
Total Litter Weight (TLW) (g)	5.755± 1.927	1.084-10.749	16.949 ± 2.916	9.022 - 28.701	343.110 ± 36.872	191.11 - 465.24	1583.305± 98.830	1305.910 - 2137.160	8854.700 ± 709.521	6467.490 - 13151.890
Mean Litter Weight (MLW) (g)	0.209± 0.057	0.099-0.410	1.553 ± 0.112	1.304 - 2.061	20.815 ± 1.603	15.923 - 25.780	129.616± 9.004	89.186 - 150.056	635.359 ± 23.984	539.381 - 759.266
Mean Within-Litter SD in Foetal Weight (g)	0.121± 0.039	0.036-0.233	0.194 ± 0.024	0.104 - 0.261	1.939 ± 0.320	0.927 - 3.038	11.571± 0.805	7.367 - 17.296	123.723 ± 16.826	40.253 - 205.243
Weight CTMLW Foetuses (g)	0.212± 0.059	0.100-0.422	1.592 ± 0.101	1.334 - 2.058	20.793 ± 1.577	15.920 - 25.660	127.934± 10.362	86.96 - 150.24	620.125 ± 24.866	544.88 - 756.75
Weight Lightest Foetuses (g)	0.069± 0.014	0.043-0.120	1.239 ± 0.103	0.901 - 1.661	17.827 ± 1.849	11.240 - 23.890	112.229± 11.282	45.76 - 128.71	377.519 ± 36.294	532.75 - 248.95
Percentage	n/a	n/a	65.387	50 -	47.009 ±	30 - 80	52.244 ±	33.333 -	54.528 ±	33.333 -

**Males in Litter (%)**		± 5.356	83.333	9.177		6.462	72.222	5.310	76.923

Abbreviations Used: SD = Standard Deviation. Ovulation Rate = Number of *Corpora Lutea* Present. Prenatal Survival (%) = (Number of Live Foetuses/Ovulation Rate) x 100. CTMLW = Closest to Mean Litter Weight foetus. PMSG=Pregnant Mare Serum Gonadotrophin. n/a = not applicable.
