# Supplementary material for: Association of foetal size and sex with porcine foeto-maternal interface integrin expression
Source: Reproduction. 2019 Jan 16;157(4):317–28. doi: 10.1530/REP-18-0520 (PMC6391912; doi:10.1530/REP-18-0520)
Supplement: Supplementary Table 2 [file supplementary_table_2.pdf]

Supplementary Table 2: Summary of RNA Quality Assessment

GD	Placenta		Endometrium	
	260/280 Mean	RINe Mean; Range	260/280 Mean	RINe Mean; Range
**18**	n/a	n/a	2.13	8.63; 7.8-9.3
**30**	2.04	7.83; 7.2-9.0	2.06	7.74; 7.0-8.3
**45**	2.08	7.12; 6.5-8.5	2.07	7.80; 7.1-8.6
**60**	2.02	7.34; 6.9-7.8	2.04	7.64; 7.0-8.5
**90**	2.04	7.04; 6.5-8.0	2.02	7.80; 6.6-8.5

GD=Gestational Day. 260/280 ratio generated spectrophotometrically using the Nanodrop ND-1000 (Labtech International Ltd.). RINe=RNA integrity number equivalent generated using the Tapestation 2200 (Agilent Technologies).
