# Supplementary material for: Association of foetal size and sex with porcine foeto-maternal interface integrin expression
Source: Reproduction. 2019 Jan 16;157(4):317–28. doi: 10.1530/REP-18-0520 (PMC6391912; doi:10.1530/REP-18-0520)
Supplement: Supplementary Table 3 [file supplementary_table_3.pdf]

Supplementary Table 3: Primer Sequences for qPCR of Candidate Genes.

Gene Symbol	Accession Number		Primer Sequence (5'→ 3')	Amplicon Size (bp)	Reference
ITGα2	NM001244272.2	Fwd	CATGCCAGATCCCTTCATCT	153	Frank *et al.*, 2017
		Rev	CGCTTAAGGCTTGGAAACTG		
ITGαV	NM001083932.1	Fwd	CGAGGACTTTGGGAATGGTTT	111	King *et al.*, 2011
		Rev	CAGTGGCAGCGACAGAAAATC		
ITGβ1	NM213968.1	Fwd	CTGCGAGTGTGATAATTTCAACTGT	112	King *et al.*, 2011
		Rev	GAACAGTCACAGGCGCTGC		
ITGβ3	NM214002.1	Fwd	TGATGCCATCATGCAGGCTAC	123	King *et al.*, 2011
		Rev	CTGCCAGCCTTCCATCCA		
ITGβ5	NM001246669.1	Fwd	GAACGAGGCCAACGAGTACAC	101	King *et al.*, 2011
		Rev	CAAAGATGAGGTTGATGTTGTT		
ITGβ6	NM001097423.1	Fwd	TCCAGCTGATCATCTCAGCTTATG	147	King *et al.*, 2011
		Rev	TCATGTGAGAGCATTTCTTTTGGT		
ITGβ8	NM001097424.1	Fwd	AATACTGTGAAAAGGATGACTTTTCTTGT	109	King *et al.*, 2011
		Rev	CCTTCCCAGCCCCTGAAG		
SPP1	X16575	Fwd	TTGGACAGCCAAGAGAAGGACAGT	121	Hernandez *et al.*, 2013
		Rev	GCTCATTGCTCCCATCATAGGTCTTG		
FN	AY839862.1	Fwd	CGGGAGGAAAAGGACAGTTCA	82	n/a
		Rev	GCCAGGAAGCTGAATACCGT		

TBP1	DQ845178	Fwd	AACAGTTCAGTAGTTATGAGCCAGA	153	Nygard *et*
		Rev	AGATGTTCTCAAACGCTTCG		*al.*, (2007)
HPRT1	DQ845175	Fwd	GGACTTGAATCATGTTTGTG	91	Nygard *et*
		Rev	CAGATGTTTCCAAACTCAAC		*al.*, (2007)
TOP2B	NM001258386.1	Fwd	AACTGGATGATGCTAATGATGCT	107	Erkens *et*
		Rev	TGGAAAAACTCCGTATCTGTCTC		*al.*, (2006)
YWHAZ	DQ845179	Fwd	TGATGATAAGAAAGGGATTGTGG	203	Nygard *et*
		Rev	GTTCAGCAATGGCTTCATCA		*al.*, (2007)

Abbreviations: bp=base pairs; ITG=integrin subunit; SPP1=Secreted Phosphoprotein 1; FN=Fibronectin; TBP1=TATA box binding protein; HPRT1=Hypoxanthine phosphoribosyltransferase 1; TOP2B= Topoisomerase II beta; YWHAZ=Tyrosine 3-monooxygenase/tryptophan 5-monooxygenase activation protein, zeta polypeptide; fwd=forward; rev=reverse. N/A indicates that the primer pair were designed specifically for this experiment using NCBI Primer Blast.
