# Supplementary material for: Association of foetal size and sex with porcine foeto-maternal interface integrin expression
Source: Reproduction. 2019 Jan 16;157(4):317–28. doi: 10.1530/REP-18-0520 (PMC6391912; doi:10.1530/REP-18-0520)
Supplement: Supplementary Table 4 [file supplementary_table_4.pdf]

Supplementary Table 4: Quantitative polymerase chain reaction calibration curve data.

Gene	Placenta				Endometrium			
	Slope	Intercept	Amplification Efficiency (%)	RSq	Slope	Intercept	Amplification Efficiency (%)	RSq
ITGα2	-3.512	22.055	92.6	0.991	-3.578	23.527	90.3	0.996
ITGαV	-3.533	25.653	91.9	0.991	-3.434	24.742	95.5	0.991
ITGβ1	-3.460	26.475	94.5	0.993	-3.224	28.247	104.2	0.991
ITGβ3	-3.548	29.146	91.4	0.994	-3.547	27.323	91.4	0.992
ITGβ5	-3.574	26.433	90.4	0.996	-3.553	27.791	91.2	0.992
ITGβ6	-3.568	28.043	90.7	0.995	-3.518	28.421	92.4	0.991
ITGβ8	-3.566	30.511	90.7	0.994	-3.524	28.012	92.2	0.995
SPP1	-3.399	20.560	96.9	0.993	-3.376	15.788	97.8	0.991
FN	-3.588	22.220	90.0	0.996	-3.571	27.152	90.6	0.991
TBP1	-3.261	27.199	102.6	0.991	-3.385	28.393	97.4	0.990
HPRT1	-3.335	26.933	99.5	0.991	N/A	N/A	N/A	N/A
TOP2B	N/A	N/A	N/A	N/A	-3.334	28.671	99.5	0.990
YWHAZ	N/A	N/A	N/A	N/A	-3.513	21.871	92.6	0.990

Gene abbreviations: Abbreviations used: ITG=integrin subunit; SPP1=Secreted Phosphoprotein 1; FN=Fibronectin; TBP1=TATA box binding protein; HPRT1=Hypoxanthine phosphoribosyltransferase 1; TOP2B=Topoisomerase II beta; YWHAZ=Tyrosine 3-monooxygenase/tryptophan 5-monooxygenase activation protein, zeta polypeptide, n/a=not applicable.
